# THE POWER OF EPHEMERAL GESTURES: THEORIES OF GENDER AND PERFORMANCE

**DOI:** 10.1093/geroni/igae098.0748

**Published:** 2024-12-31

**Authors:** Nels Highberg, Emma Chamberlin

**Affiliations:** University of Hartford, Hartford, Connecticut, United States; University of Connecticut, Storrs, Connecticut, United States

## Abstract

Questions of what defines gender have long been central to theorists, activists, and artists who place gender and sexuality at the center of their work. This presentation provides an overview of feminist and queer theories that can deepen the thinking of those who engage with drag performers. We begin by providing a definition of cultural hegemony informed by the work of Antonio Gramsci and James Lull. Theorizing of power and ideology – including ageism, sexism, and homophobia – Gramsci and Lull argue that dominant ideologies result from intergroup interactions like those exemplified in the anti-drag legislation that dominates the United States. From there, we shift to feminist and queer theories of performativity and identity formation. We start with the foundational work of Judith Butler, whose “Gender Trouble: Feminism and the Subversion of Identity” in 1990 and “Who’s Afraid of Gender?” in 2024 ask how much of “gender” comes not from an essential, internal core but from repeated, unconscious actions enforced by external socio-political forces. That leads to the value of performance and gesture in the creation of a lasting queer legacy. José Esteban Muñoz theorized a queer ephemera that includes drag in his 2009 publication of Cruising Utopia: The Then and There of Queer Futurity, highlighting how gestures in drag aid in the creation of a culturally forbidden queer history, a history that becomes richer and more complex as these drag artists – along with theories about gender and sexual minorities in general – age.

